# Physical environmental influences on human health

**DOI:** 10.3205/dgkh000599

**Published:** 2025-11-28

**Authors:** Katrin Steul, Sabine Meyer, Yanfeng Xue

**Affiliations:** 1 Public health Authority, Offenbach District, Dietzenbach, Germany; 2 KLW St. Paulus GmbH, St. Marien Hospital Lünen, Lünen, Germany

**Keywords:** environmental medicine, environmental health, physical environmental, noise, particulate matter, ultrafine particles, radiation, heat

## Abstract

This review examines physical environmental influences and their potential effect on human health. The factors discussed are noise (effect of sound), the physical effect of particulate matter (fine dust, particulate matter PM10, PM2.5, ultrafine particles UFP), various types of radiation (electromagnetic fields, radioactivity) and climate (especially heat). In order to identify hazard potentials, it is necessary to know the route of exposure and the mode of action. Environmental epidemiology studies help to illustrate the health consequences for the population. The limitations of the studies must also be considered.

## Abstract (de)

Dargestellt werden physikalische Wirkungen von Lärm (Wirkung des Schalls), Feinstaub (luftgetragene Partikel), Strahlung (elektromagnetische Felder, Radioaktivität) sowie Klima (insbesondere der Faktor Hitze) auf die Gesundheit des Menschen. Zur Abschätzung von umweltmedizinischen Gefahrenpotenzialen werden die Wirkweise der Faktoren sowie der wahrscheinliche Expositionsweg benötigt. Studien aus der Umweltepidemiologie helfen, die gesundheitlichen Folgen für die Bevölkerung zu bewerten. Diese unterliegen immer auch gewissen Limitationen, die bei der Bewertung mit zu beurteilen sind.

## Introduction

Environmental medicine is generally referred to as “human medical impact research”. It investigates which environmental factors, either alone or in combination, influence the health and/or the well-being of humans [1], [2], [3], [4], [5], [6]. There are different definitions of the terms environmental medicine and environmental health. However, these definitions repeatedly refer to similar aspects that characterize environmental medicine; these include:

- Defining itself as an interdisciplinary field. The disease patterns span various medical disciplines and may affect all organ systems of the human body.
- Distinguishing the following tasks from each other: The clinical diagnosis and treatment of individuals versus the assessment of environmental hazards for the entire population (individual medicine versus public health).
- Dealing with exposures and their effects on human health.

Environmental medical exposures can be subdivided into

- Chemical substances/substance groups,
- biological agents and
- physical influences.

Chemical substances are absorbed, for instance, as an indoor air pollutant (inhalation) or orally through the consumption of food or drinking water. Relevant substance groups include aromatic (benzene) and other hydrocarbons, which are often used as solvents or as original or intermediate chemical compounds for other chemical products (perchloroethylene, formaldehyde, pentachlorophenol [PCP] etc.). Toxic effects following exposure are also attributed to metals (such as aluminum, lead, nickel etc.), inorganic gases (e.g., ozone, sulfur dioxide, etc.) and various types of biocides. 

Biological agents such as pollen or molds are usually absorbed through inhalation. Certain plants or animals may trigger allergic reactions (oak processionary moth, ragweed), for example, after skin exposure. This group of agents must also be assessed in terms of medical issues, as biologigal agents can be intensified by environmental influences.

This review will focus on how human health is influenced by physical factors. 

## Influences of noise on human health

Noise is commonly defined as unwanted (also individually perceived as such) sound. Sound arises from vibrations in the air (as well as in liquids and solids). It can be objectively characterized by frequency and intensity. The measure is the sound intensity level, expressed in decibels (dB). This denotes the logarithmic ratio between the measured intensity of the vibrations (or the heat flux density with the unit W/m²) and a standardized reference value. This reference value is calculated as 10–12 W/m². An increase in the sound intensity level by 10 dB then corresponds to a linear increase of the intensity by a factor of 10 [7], [8], [9], [10]. For example, a noise with a sound intensity of 80 dB therefore does not transmit twice as much energy as one with 40 dB, but 10,000 (10^4^) times as much. 

In addition to the sound intensity level in W/m², sound pressure (unit Pa) can also be used to evaluate the sound. Here, sound pressure level is determined by the logarithmic ratio between the effectively measured sound pressure and a reference value. The resulting sound pressure level is also expressed in the auxiliary unit of measurement dB.

In air, the sound intensity level (calculated by the intensity) coincides with the sound pressure level so that both terms can be used analogously in studies. It is often only referred to as the sound level.

It is known from noise impact research that average-level noise (the calculated average value of the sound level) over a time period is usually not adequately accounted for Studies indicate that annoyance depends more strongly on the maximum level. Studies on annoyance by aircraft noise in contrast to other noise events (e.g., road traffic noise, railway noise) have found that, at the same average sound level, people are more annoyed by aircraft noise probably due to the frequency and extent of the maximum levels [7]. The energy-equivalent continuous sound level (LAeq) represents the sound energy over the entire measurement period and not just the average of individual sound levels. For this reason, it is a more informative parameter. 

The hearing threshold for non-impaired hearing is at approximately 0 dB. The pain threshold is around 130–140 dB (Table 1 (Tab. 1)). The essential sources of environmental noise are traffic, industrial facilities and recreational noise. An overview of various environmental noise sources along with their sound intensity levels can be found in Table 1 (Tab. 1).

**Figure 1:**
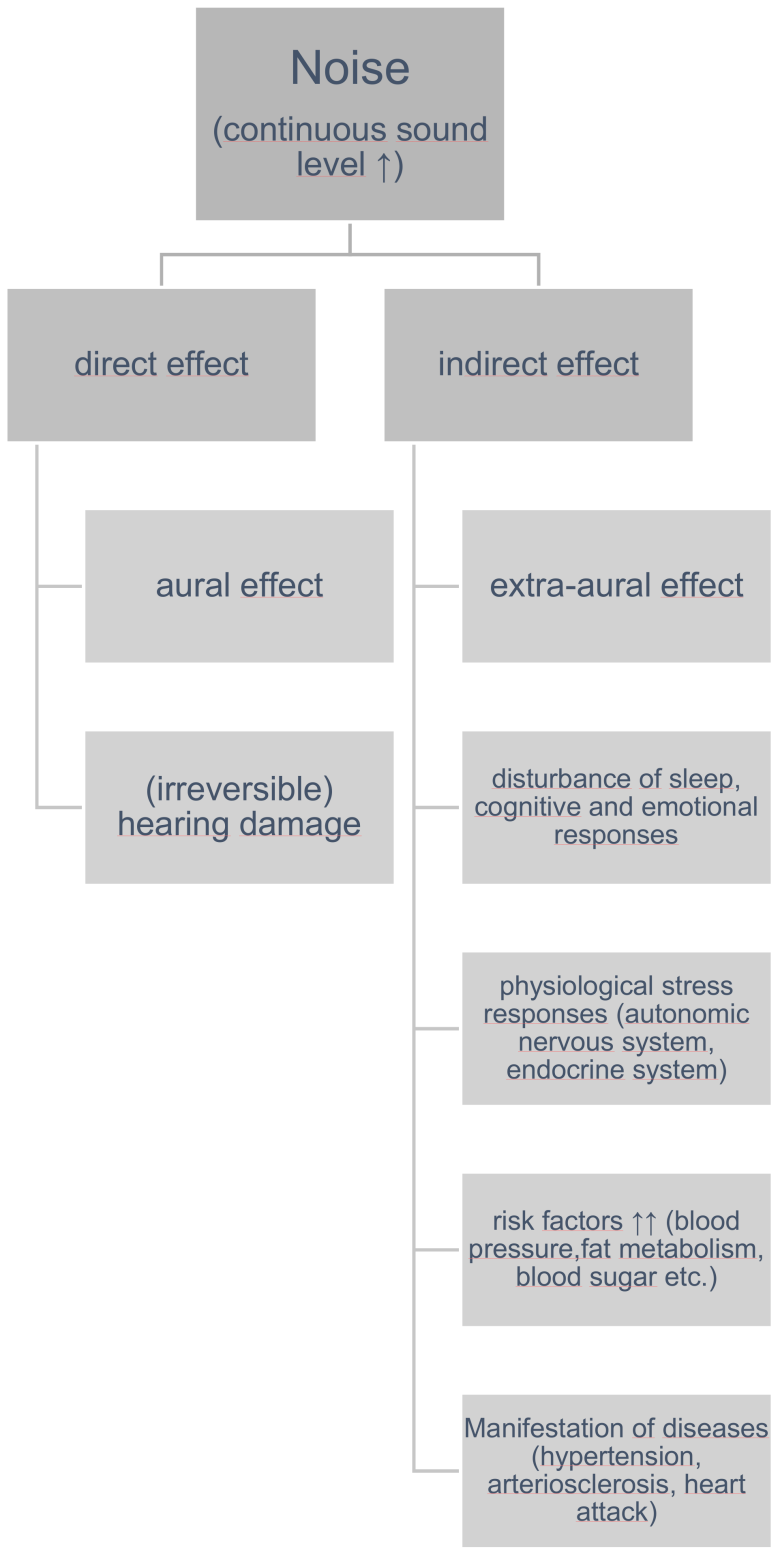
Noise impact model according to Babitsch et al. [8], [45]

In principle, noise effects can be distinguished between those that directly affect the auditory system (aural effects), and those that impact the rest of the organism (extra-aural effects). Aural noise effects with mostly irreversible damage to the inner ear occur for instance after a single very intense noise exposure (blast trauma, >140 dB) or after years of regular noise exposure (>85 dB). These long-term noise exposures are found particularly among individuals who are occupationally exposed to noise. Thus, they fall under the category of occupational health-relevant exposures. In the context of environmental physics, the effect of sound is “indirectly” mediated. It can be assumed that the majority of extra-aural disorders are caused by an unspecific activation of the autonomic nervous system and certain endocrine responses (Figure 1 (Fig. 1)).

Figure 1 (Fig. 1) illustrates the noise impact model according to Babisch: the disturbance of night sleep and other stress reactions (anger; cognitive and emotional responses to noise disturbance) lead to physiological stress reactions. These act as risk factors especially for the manifestation of cardiovascular diseases. Not only cardiovascular diseases, but also sleep disorders, cognitive impairments and mental illnesses are considered potential health consequences of significant noise exposure [7], [11].

In order to describe the connection between noise pollution (exposure) and health reaction, noise impact research uses what is known as the “exposure-response curve”. In these curves, the x-axis represents the level of noise exposure and the y-axis the corresponding response. The noise exposure is usually defined as the energy-equivalent continuous sound level over the studied period. The corresponding health reaction is defined in advance, either by the extent of impairment or by the frequency of a certain event (among the individuals studied). From studies in noise impact research, it is known that noise sources are perceived differently. For instance, residents living near airports judge the maximum noise of aircraft movements with subsequent noise breaks as particularly annoying. In constrast, road traffic noise sometimestakes on the character of white noise The perception of “neighborhood noise” (kindergarten, pedestrians, bars/restaurants etc.) is often strongly influenced by how the different noise sources are evaluated by the test subjects, for example, whether the attitude towards the studied noise source tends to be positive (“I like children. The kindergarten doesn’t bother me much.”/ “I/we like living in the city center.”) or negative. 

Extensive noise impact studies exist on the effects of road traffic, rail traffic and especially aircraft noise. Particularly from the latter it is known that both children and adolescents as well as adults feel annoyed by (aircraft) noise, albeit to a lesser extent among children (and adolescents). In some studies, the different types of traffic noise (rail, road, air traffic) were associated with an increase in cardiovascular diseases. Whether this is the case and how severe the impairment is, depends largely on the individual circumstances and the study design. Furthermore, the extensive noise impact studies (NORAH [12], [13], HYENA [14], [15], RANCH [16], [17] etc.) revealed sleep disorders, depressive disorders and in some cases cognitive impairments. The evidence of the correlation is highly dependent on the study design and the population [7], [18]. 

Recommendations can be derived from the noise impact research described as follows. WHO international guidelines for avoiding health hazards from environmental noise define the recommended maximum exposure to aircraft noise as 45 dB of equivalent continuous sound level (day and night) and 40 dB at night, to rail traffic noise as 54 dB (day and night) and 44 dB at night, to road traffic noise 53 dB (day and night) and 45 dB at night [11].

## Influence of particulate matter (airborne particles) on human health

**Figure 2:**
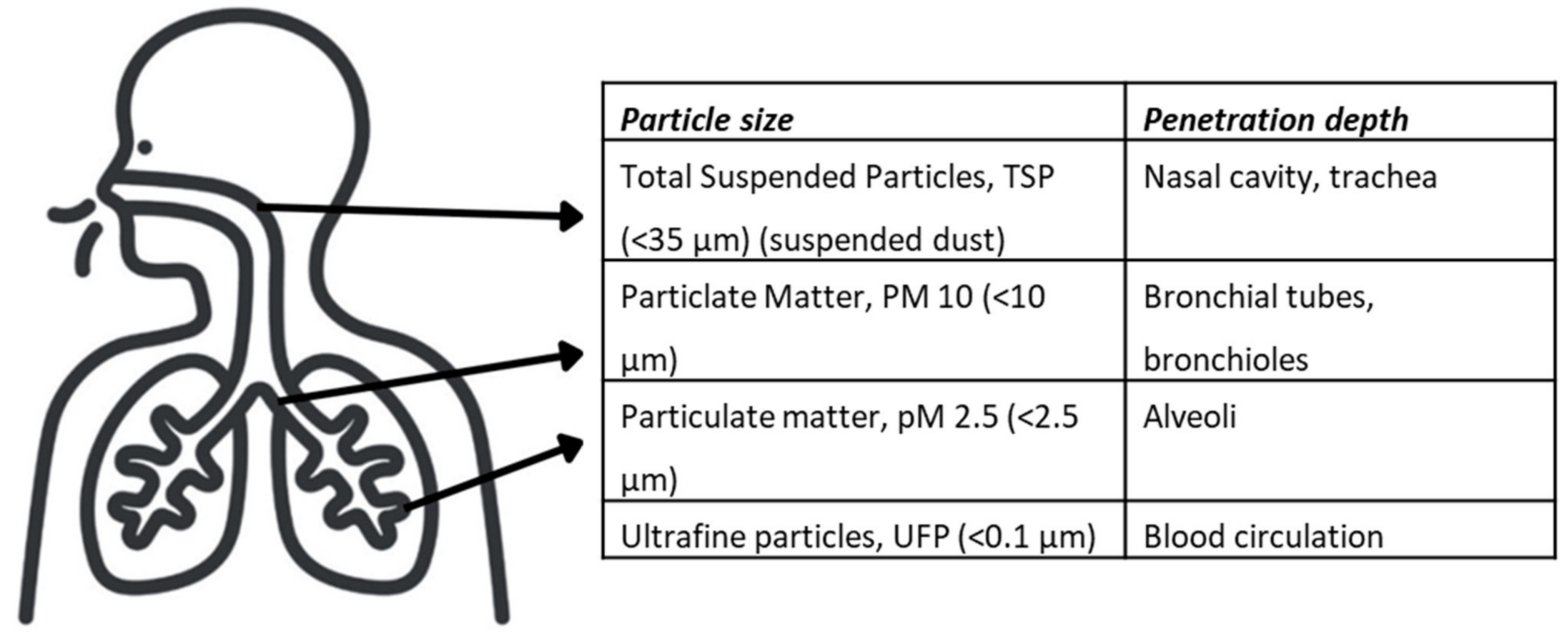
Penetration depths of particulate matter PM10, PM2.5, and ultrafine particulate matter (UFP) by inhalation [19]

Dust refers to all solid and liquid particles in the air. The particles differ from one another on the basis of their chemical and physical charasteristics (particle size and shape), which are crucial factors in terms of their health effects [19]. Depending on their size, they can penetrate to different depths into the human respiratory tract. The particle fractions therefore also differ in their respective locus of action within the respiratory tract (Figure 2 (Fig. 2)).

Particles with an aerodynamic diameter smaller than 35 µm are referred to as suspended particulate matter (total suspended particles, TSP). Due to the property of suspended particulate matter to “float in the air”, it can easily enter the lungs with the air we breathe. Particles with a size (diameter) over 10 µm remain mainly in the upper airways of the nasopharynx and the trachea. The mucous membrane of the upper airways is capable of eliminating these particles. Particles with a diameter less than 10 µm (particulate matter, PM10) penetrate deeper into the respiratory tract, reaching the bronchi and bronchioles. They are therefore referred to as inhalable airborne dust or thoracic dust. An even smaller diameter of less than 2.5 µm (particulate matter, PM2.5) allows particles to reach the alveoli. The ultrafine particles (UFP) are even smaller (<0.1 µm). These are not completely retained in the alveoli, but can reach the bloodstream.

The various size fractions of TSP, PM10, PM2.5, and UFP originate from different sources. The larger particles (TSP, PM10) tend to come from mechanical processes or have biological origin, e.g., pollen, sand, and volcanic eruptions. The mechanical processes are diverse: dust from open-cast mining or from trade and manufacturing processes as well as abrasion processes indoors, e.g., from office work. The smaller particles (PM2.5, UFP) tend to be generated during combustion processes. A significant proportion of smaller particles in particular is attributed to industrial processes or traffic [19]. During combustion processes, other factors are simultaneously produced, such as nitrogen dioxide, which is classified as an irritant gas (NO_2_). In addition, combustion processes such as occur in traffic cause emissions of different types (carbon monoxide, elemental carbon, benzene etc.). It is difficult to separate the chemical effect of these emissions from the physical effect of particulate matter. Nevertheless, there are a large number of environmental epidemiological studies that demonstrate the isolated harmful effects of PM10, PM2.5, and UFP [20], [21], [22].

A distinction can be made between short- and long-term effects. The short-term effect of increased PM exposure primarily involves impairment to the respiratory system itself. With previously damaged lungs (bronchial asthma, chronic obstructive obstructive lung diseases, etc.), a short-term increase of PM exposure leads to an impairment of lung function and an increase in treatment interventions. Some studies demonstrate an increase in cardiovascular diseases due to short-term exposure to PM. It is assumed that the particles penetrate into the deep respiratory tract that trigger ischaemia, thrombosis, and other cardiovascular events [20]. The long-term effects of PM have been less well researched. This is also due to the fact that environmental epidemiological studies always have to account for the simultaneous influence of various factors, and it is methodologically challenging to clearly isolate these effects from other influencing factors (e.g., NO_x_, carbon monoxide, noise). 

In response to the existing environmental epidemiological studies on the effect of PM on health, international and national guidelines have been developed [23], [24]. Currently, a guideline limit of 15 µg/m³ PM10 is established as an annual mean [24]. For PM2.5, there is a guideline limit of 5 µg/m³ as an annual mean. The EU limits, frequently discussed in public debates related to air quality management plans, are currently set at 40 µg/m³ for PM10 (annual mean) and 25 µg/m³ for PM2.5 (annual mean) (Table 2 (Tab. 2)). The principle from both the EU limits as well as the WHO guideline limits is that average values must be adhered and individual peaks should be limited. For this reason, daily average values have also been established, which may only be exceeded on a few days per year (Table 2 (Tab. 2)).

## Influence of radiation (ionizing and non-ionizing) on human health

### Radiation

**Figure 3:**
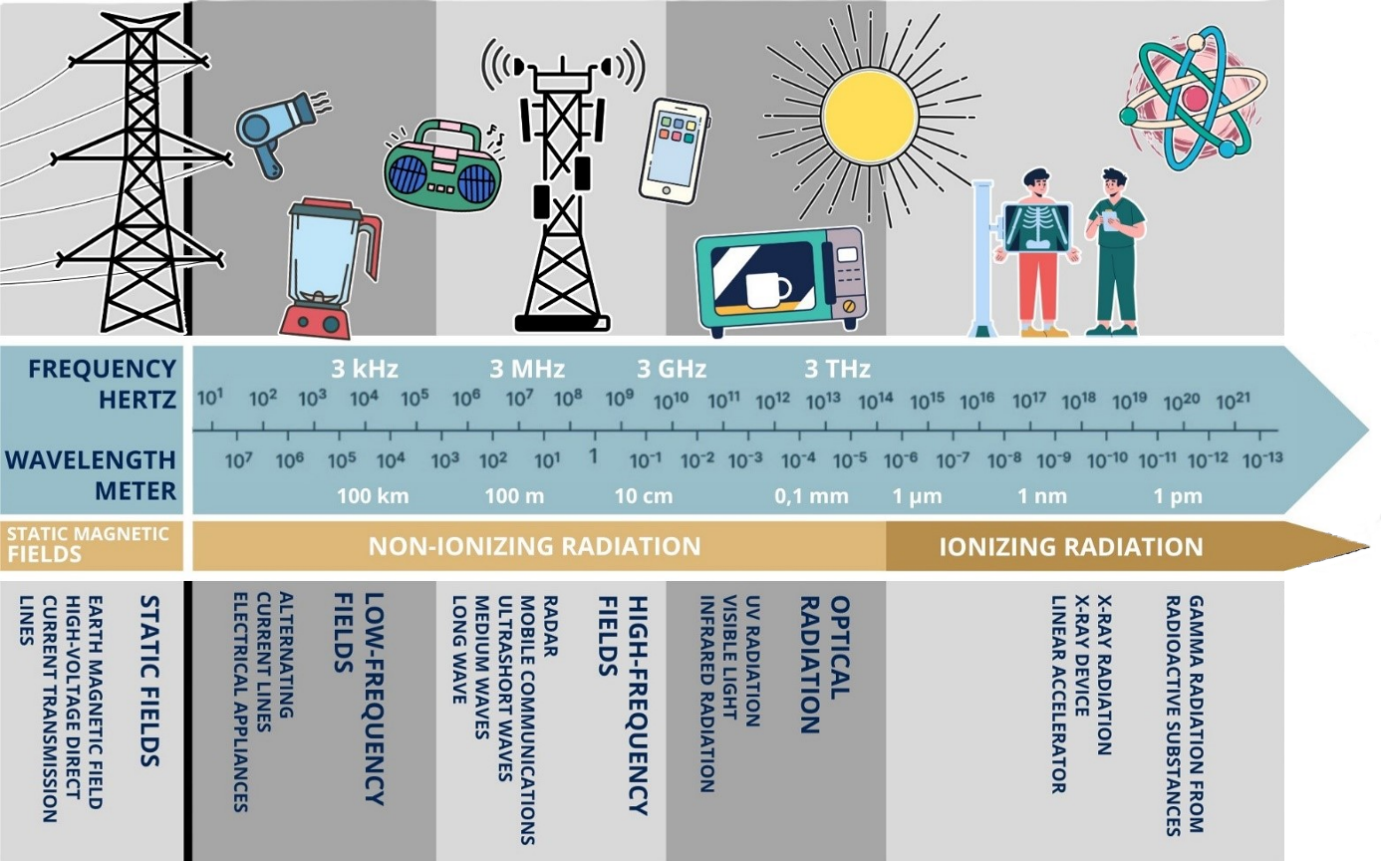
Frequencies and wavelengths of non-ionizing and ionizing radiation [10]

Depending on the energy, a distinction is made between ionizing and non-ionizing radiation. In the case of non-ionizing radiation, the energy is not sufficient to convert atoms or molecules into an electrically charged state (they are not ionized). Therefore, non-ionizing radiation is not radioactive. Additionally, static fields with magnetic effects, such as the Earth’s magnetic field, must be differentiated from non-ionizing radiation Magnetic fields are generated by moving electric charges in direct current (at 0 Hz), in contrast to alternating current in the low frequency range of non-ionizing radiation. Non-ionizing radiation is further divided into several overlapping ranges based upon wavelength and frequency. These include the high-frequency ranges of optical radiation (UV radiation, infrared, etc.), the high-frequency radiation emitted by mobile phones, microwaves etc. and the low-frequency fields of electrical devices (Figure 3 (Fig. 3)). 

### Electromagnetic fields (EMF)

The non-ionizing radiation range with frequencies between 0 and 300 GHz is also referred to as electromagnetic fields. High-frequency and low-frequency fields have different physical functions and thus affect the human body in different ways. Low-frequency fields (0–9 kHz) influence the body’s own electrical currents and therefore have a potentially stimulating effect on nerve and muscle cells [25]. For instance, a metallic taste is described at <1 Hz, flashes of light at approx. 20 Hz, and stimulation of individual muscle fibers at approx. 50 Hz [10]. High-frequency fields (9 kHz–300 GHz) have the potential to penetrate a few centimetres into the body. They are absorbed and converted into heat. At a certain intensity, cells can be damaged by the heat effect (from approx. 100 kHz). Compared to the irritating effect of the low-frequency fields, thermal effects occur in the higher frequency range. The ranges merge into one another [10]. 

The potential health risks associated with the electromagnetic fields (high and low frequencies) mentioned are often referred to as “electrosmog” in the media and in public discourse. The term “smog” is derived from the words “smoke” and “fog”. It generally refers to the presence of air pollutants harmful to human health [26]. Although electromagnetic fields are also natural phenomena (Earth’s magnetic field, thunderstorms, lightning, UV radiation). However, “electrosmog” typically refers to only those sources of exposure that are anthropogenic.

To assess the potential risks of anthropogenic electromagnetic fields (EMF), there are different approaches. The same is true for causality research with other environmental hazards. For instance, in-vitro studies on cell or tissue cultures can serve to demonstrate the mechanism of action of a certain radiation exposure. However, whether effects are relevant to health and could lead to a specific disease cannot be determined with this study design. In animal studies, the effects on entire organ systems can be examined under standardized conditions and with random assignment to an exposure group. Nevertheless, the results cannot be directly transferred to humans and lower levels of exposure in everyday life. In human studies under standardised condition, acute effects or physiological reactions to a defined radiation dose can be investigated. However, this study design refers to an acute effect of a low, short-term radiation exposure. In observational epidemiological studies of large groups of people, peoples are examined regarding the probability of developing defined symptom complexes. In such studies, the actual radiation exposure in individual cases can only be roughly estimated. Furthermore, individuals cannot be randomly assigned to a group and are also exposed to other factors. In high-quality studies, these can be partially controlled (provided influencing factors are known). Unlike intensively studied noise-impact research, only a few studies on low-level radiation are available that allow unambiguous conclusions [26]. These are used to set limit values for operation of various systems (high frequency, low-frequency, direct current systems) (German Regulation on EMF [27]). 

Exposure to ionizing radiation refers to natural (cosmic rays, terrestrial radiation sources) as well as anthropogenic sources (e.g., from nuclear fission). The causes of anthropogenic radiation exposure in Germany range from radionuclides, which are associated with ventilation or wastewater from nuclear facilities, to the excretion y persons receiving radioactive substances for therapy or diagnostics [28]. The human body is usually able to cope with ionizing radiation from natural radiation sources. However, ionizing radiation, regardless of whether it is of natural or anthropogenic origin, has potentially damaging effects on cells as the smallest biological unit, by altering or damaging the genetic material contained therein. However, the extent of radiation does not exactly correspond to the occurrence of health damage. The human organism has the ability to recognize and repair damaged cells or, after the cell death, to restore the organ system to an undamaged state. Health damage is more likely to occur when a very large amount of radiation affects the organism over a short period [10]. Two fields of action of ionizing radiation on human health organisms can be distinguished. First, the deterministic radiation effect is that which can be directly attributed to a usually high radiation dose. It occurs immediately or shortly after radiation exposure when a critical number of cells are damaged. This effect occurs in humans at a dose of at least 500 mSv (0.5 Sieverts). Without medical intervention, exceeding this threshold by more than 10-fold usually leads to death in humans [10]. Second, the stochastic radiation effect is defined as an increased probability of health damage after several years or decades of exposure. The radiation dose does not affect the severity of expected radiation damage, but rather the probability of occurance. This relates to a random radiation effect: DNA is damaged by radiation and this information cannot be repaired; it is thus passed on to subsequent generations of cells. Changes occurring here can lead to genetic variations or malignant cancer. The probability of a stochastic radiation effect is called the risk of damage or the radiation risk [10]. To protect the population from ionizing radiation, protection from naturally occurring radon, occupational and medical radiation protection, as well as inspection of nuclear facilities, are regulated by law in Germany in the radiation protection ordinance [29].

## Influence of climate on human health

Due to the changes in global climate during the past several decades, effects on public health might be expected. The term “climate” refers to the average state of the atmosphere in a given region over a longer period of time (at least 30 years) [30]. Characteristic extreme values and frequency distributions are also considered in the definition. Thus, climate changes can be defined as follows:

- Change in the average temperature over time: the annual global mean temperature in relation to the global average temperature during a reference period. Compared to the reference period from 1961–1990, the global mean temperature has lately increased by approximately 1.0°C, and in Germany by 1.5°C [31].
- Days with special temperature maximums: they can be compared to the days with same definition during a reference period. For example, in Germany during the reference period 1961–1990, 4.2 “hot days” per year were recorded. Up to 2018, an increase in “hot days” of more than 7.3 days can be assumed since 1951 [32].
- Climate always refers to a specific geographical location: the continental climate with strong daily as well as seasonal temperature fluctuations (e.g., in Moscow) differs strongly from that of maritime climates (such as Dublin). In the maritime climate zone, the temperature is rather stable, as areother weather elements such as precipitation. Changes in these variation ranges are also an indication of climate change.

Climate change can have natural and anthropogenic causes. Anthropogenic causes include the burning of fossil fuels (coal, natural gas, etc.) and changed land use through expansion of livestock farming. These lead to an increase in carbon dioxide, methane, nitrous oxide, and other gases. All of these substances, including water vapor and ozone from natural sources, allow the sun’s rays directed at the Earth to pass through the atmosphere unimpeded, but absorb the long-wave radiation emitted by the Earth’s surface. This heat radiation is emitted in all spatial directions, in part including back to the Earth’s surface (thermal counter-radiation). It leads to an additional warming of the Earth’s surface, which is known as the greenhouse effect. The impact of the greenhouse effect is immense. Without it, life on Earth would not be possible. The currently enhanced anthropogenic greenhouse effect leads to climate change. 

The term “heat” is understood to mean high temperatures, which are perceived as unpleasant and cause a health burden. The burden of heat reduces both the physical resilienceas well as physical performance. Physiologically, this can be explained by the following two mechanisms:

- Vasodilation improves heat release from the muscles to the skin surface.
- Perspiration on the skin leads to cooling of the body surface.

These regulatory mechanisms are controlled by the central nervous system, namely through temperature-sensitive receptors on the skin surface. They are necessary to prevent overheating of the body core temperature. Understanding these mechanisms explains the various impairments caused by heat: The redistribution of blood volume by vasodilation increases the oxygen demand of the heart and simultaneously impairs the heart’s pump function due to insufficient filling. This can lead to cardiac ischemia and infarctions, particularly in individuals with pre-existing diseases. Perspiration leads to water and electrolyte deficits and often to dehydration, which can also have numerous cardiovascular effects [33]. 

Many national [34] and international studies confirm the increased mortality associated with high outdoor temperatures [35], [36]. The challenge lies in attributing individual deaths to a specific heat event. In an extrodinary heat situation, people may die due to pre-existing underlying conditions or accidents caused by weakness, but these deaths might be recorded as results of underlying condition or the accident. To include these deaths in statistics, the overall mortality is used to correlate them with days with a temperature defined as “heat”. The effect of increased mortality in heat can be observed in almost all climate zones [36]. Comparative studies indicate the strongest effects of heat on mortality occur in South and South-East Asia, North Africa and Sub-Saharan regions as well as in the Middle East [36]. In numerous studies on heat-related mortality, this effect has also been reliably demonstrated for Germany and Central Europe [37], [38], [39]. 

In the analysis of morbidity, there are different approaches regarding which cases of diseases to be considered and how the data are collected. Not only the diagnoses that explicitly reference heat (such as heat stroke, heat exhaustion, etc.) are relevant, but also other cases of illness that are related to the heat responses of the human body described above. In a person with a pre-existing disturbance of water balance/chronic disease (kidney disease, diuretic drugs for hypertension, etc.), a heat situation with increased perspiration can lead to an ischemic event. Various accident scenarios in response to heat are possible. Therefore, in international studies, total cases of admissions to emergency services or acute hospital admissions are used to estimate the morbidity [40]. Significant effects are usually observed on days with exordinary heat. In Germany, there is limited research on the increase in morbidity due to heat. Available research also shows a correlation between hospital admissions via emergency services and heat [41], [42].

The definitions for days or situations with a special heat load vary widely internationally and nationally. For terms such as heat or heat wave etc. there is not a standardized efinition. The definition of a heat event is often based on the maximum daily temperature. “Heat waves” are usually referred to as several consecutive days with maximum temperatures above a specified threshold [40], [43]. The German Weather Service applies the concept of “perceived temperature”, which is used to warn the population in a region in Germany about extreme heat. In addition to temperature, other meteorological factors such as humidity, the amount of precipitation, or wind speed are taken into account to define the perceived temperature. The minimum night temperature describes cooling during the night.

Climate has a significant impact on human health. The best-researched parameter is heat (high temperatures). Other climate parameters, however, must be taken into account when assessing health risks. Since the climatic conditions of different regions are not comparable, epidemiological studies on the environmental factor "climate" must always consider the regional specifics.

In addition to the physical effects, climate change also impacts the transmission probability of infectious diseases. The shift towards warmer temperatures favors the spread of disease vectors. The transmission of vector-associated pathogens requires suitable climatic conditions throughout the entire cycle of pathogen transmission. Globalisation brought *Aedes* *albopticus* (Asian tiger mosquito) to Europe, but only the higher annual temperatures lead to relevant populations, which can act as vectors for Dengue or Chikungunya virus. Higher temperatures in combination with sufficient humidity accelerate the developmental processes of ticks (e.g., *Ixodes* *ricinus*/the common wood tick), which can transmit infectious agents such as FSME virus or *Borrelia* in Central Europe. These indirect climate impacts require monitoring [44].

## Conclusions

To assess environmental hazards, exposure pathways and mechanisms of action must be known. Epidemiological research helps to illustrate the health consequences for the population. However, the methodical limitations of research must also be taken into account.

## Notes

### Competing interests

The authors declare that they have no competing interests.

## Figures and Tables

**Table 1:**
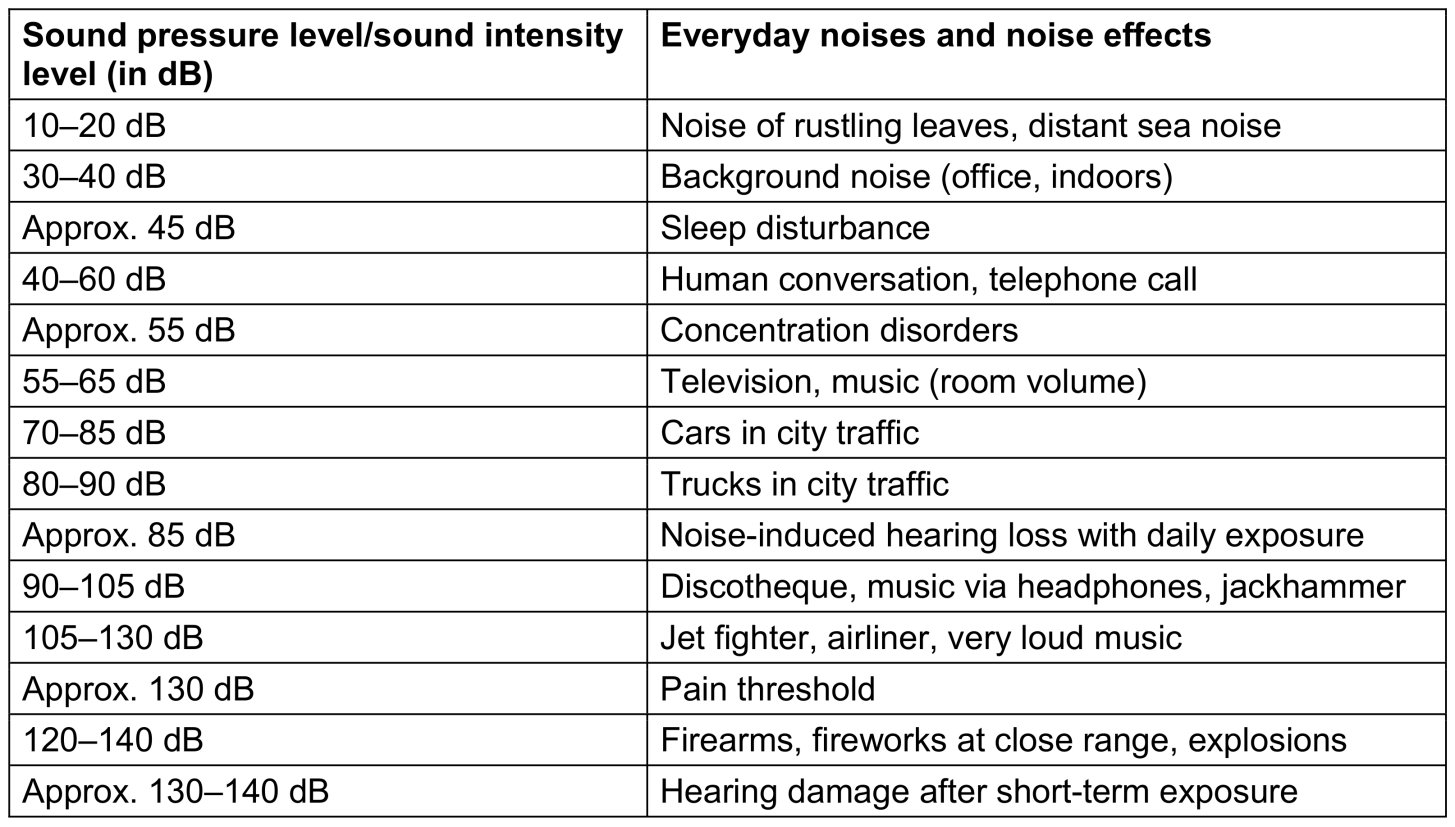
Sound intensity level of everyday noises and noise effects

**Table 2:**
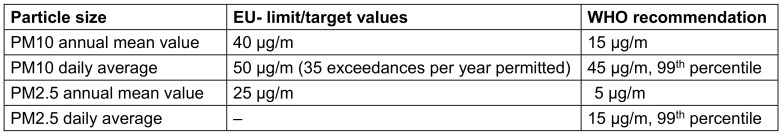
Limit/target values of the EU directive [23] and the WHO recommendations [23]
